# Supplementary material for: Removal of 4-chloro-2-methylphenoxyacetic acid from water by MIL-101(Cr) metal-organic framework: kinetics, isotherms and statistical models
Source: R Soc Open Sci. 2021 Jan 13;8(1):201553. doi: 10.1098/rsos.201553 (PMC7890509; doi:10.1098/rsos.201553)
Supplement: Suplimentary materials [file rsos201553supp5.docx]

Statistical Tool

Link: jmp.com/en_us/download-jmp-free-trial.html
